# Compositional differences of milk from pasture-based systems compared with systems consuming large amounts of non-pasture feeds

**DOI:** 10.3168/jdsc.2026-0987

**Published:** 2026-04-20

**Authors:** Jonathan B. Magan, John T. Tobin, Tom F. O'Callaghan, Noel A. McCarthy, Sean A. Hogan, Mark A. Fenelon

**Affiliations:** 1 Food Chemistry & Technology Department, Teagasc Food Research Centre, Moorepark, Fermoy, Co. Cork, Ireland, P61C996; 2 School of Food and Nutritional Sciences, University College Cork, Cork, Co. Cork, Ireland, T12Y337; 3 Teagasc Food Research Centre, Moorepark, Fermoy, Co. Cork, Ireland, P61C996

## Abstract

Pasture-based milk production systems represent a considerable minority from a global perspective but encompass unique management factors, which lead to characteristic differences in milk composition relative to conventional production systems. Synchronized calving patterns, associated stage of lactation effects and coincident variation in dietary composition throughout the year contribute to seasonal changes across a range of compositional factors from early transition milk to mid-lactation and late-lactation milk. The most substantial and identifiable impacts of pasture-based production are resolved in the fatty acid profile of milk; however, considerable shifts in gross compositional factors, nitrogen composition, mineral and vitamin profiles, and minor volatile compounds and metabolites also present seasonal trends and notable changes in concentration in response to grass feeding.

## Highlights

• Dietary factors significantly influence the macro- and micro-composition of milk.
• Sward composition is also an important factor that leads to compositional variation.
• The fat fraction is most susceptible to alterations in diet.
• Compositional differences have been shown to alter product sensory characteristics.
• Specific biomarkers linked to feed types can be used for product verification purposes.

## Graphical Abstract

Across numerous studies, significant compositional variation has been observed between milk derived from pasture-based and non-pasture-based feeding systems. At a gross compositional level, pasture-based feeding systems have been widely reported to be associated with significant increases in percentage total solids, fat and protein, whereas total mixed ration (TMR) systems have been associated with significantly higher milk yields and lactose percentages. Pasture-derived milk has also shown significantly higher concentrations of n-3 fatty acids, whereas increases in n-6 fatty acids have been associated with TMR systems. Differences in the micronutrient composition of milk have also been observed between systems. Pasture feeding has been associated with increases in concentrations of fat-soluble vitamins and the water-soluble vitamins B2 (riboflavin) and B7 (biotin). Milk from TMR systems has been associated with increased concentrations of trace elements such as iodine and selenium and the water-soluble vitamin B3 (niacin).

**Figure:**
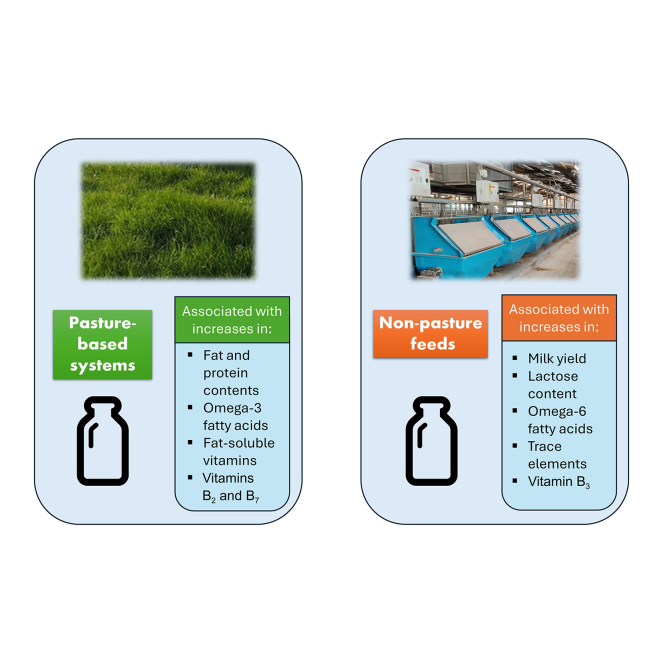

Ireland's competitive advantage and efficiency in milk production is derived from maximization of utilizing pasture as the primary feed source. Ruminants, due to their complex, multicompartmental digestive systems, can efficiently convert the non-human-edible materials in grass into highly nutritious products suitable for human consumption. A wide range of research has demonstrated the points of difference between milk and dairy products derived from pasture-based systems compared with milk from conventional indoor TMR feeding systems, which will be outlined in this review. In the case of cow diet, it is important to define pasture, as it is not only grass (a term which may also include silages), but also includes a variety of plants or forages grazed outdoors, including but not limited to rye (*Lolium*) and other grass species, as well as white clover (*Trifolium* *repens*), red clover (*Trifolium pratense*), plantain (*Plantago lanceolata*), chicory (*Cichorium intybus*), herbs, and a variety of other plants present at varying frequencies of distribution within a sward.

When it comes to true pasture systems that maximize potential DIM with cows grazing outdoors, seasonal milk production is the norm, where the stage of lactation of the milking herd follows the growth patterns of grass and forage, providing an efficient conversion of grazed forages into milk. This economically incentivized synchronization between the milk production window and the annual pattern of grass growth necessitates that most cows calve within a narrow window (e.g., late winter and spring period) and results in variability in dietary composition throughout the year. The combination of dietary and lactational changes, as the majority of cows progress through their lactation cycles in a synchronized pattern, results in a distinctly seasonal milk supply and consequent variability in milk composition and functionality.

Because the stage of lactation is intrinsically linked to diet in a seasonal grass-based production system, its effects on milk composition throughout the year warrant some discussion. Milk from very early lactation and late lactation is characterized by elevated total solids content due to increases in total protein, fat, and ash contents, which exceed the decline in lactose content (Li et al., 2019), occurring in response to an increase in osmotically active salts within the overall elevated ash content (Fox et al., 2016). Moreover, the mineral profile shifts throughout the season (Loveday et al., 2021), including particular increases in sodium and total, serum, and ionic calcium. These changes, in combination with a proportional increase in whey relative to casein, and increases in SFA, pH, and enzymatic activity, render late-lactation and very-early-lactation milk less suitable for processing under standard conditions, leading to challenges for milk processors relating to butter hardness, cheese clotting and yield, and the heat stability of milk during thermal treatments. The elevation in total solids, and particularly protein content, also necessitates a greater degree of standardization using lactose or milk permeate in products such as skim milk powder, as the protein/lactose ratio varies throughout the season. These phenomena result in a distinctly seasonal product portfolio focused on long-shelf-life products such as butter, cheddar cheese, and milk powders. Although this may pose challenges for manufacturers associated with winter milk volumes, capital usage, and seasonal alterations to processing parameters, as of 2020, this largely synchronized spring calving system has been shown to be optimal for the majority of Irish farmers, in comparison to a split-calving “winter milk” system. Availing of the low cost and plentiful supply of grass across a ~300 d grazing season, followed by a dry period, is estimated to represent €128 million in profitability for Irish dairy farmers in comparison to a projected shift toward a 50:50 spring/autumn calving system (Shalloo et al., 2020).

Colostrum is the first milk available to the calf following birth, with transition milk produced over subsequent days (second to sixth milkings), after which it is compositionally similar to mature milk (O'Callaghan et al., 2020). Colostrum accounts for approximately 0.5% of annual milk output. The composition of colostrum and subsequent transition milk is affected by numerous factors, including maternal nutrition, breed, parity, length of the dry period before calving, and the time of milk collection postpartum (McGrath et al., 2016; Masterson et al., 2024). Within spring calving systems, colostrum and transition milk are generated over a window of ~6 weeks, typically from late January to March in Ireland, coinciding with the start of the grazing season. The vast majority of work carried out to date on the effects of pasture-based milk production on milk composition has been conducted on mature milk, with relatively little information available on precursor colostrum and transition milk.

In comparison with mature milk, colostrum has a higher total solids content (~27%) and contains ~5 times more protein (~15%), which decreases during the transition period (McGrath et al., 2016). The ratio of whey to casein proteins in colostrum (~70:30) is significantly higher than that of mature milk (20:80). McDermott et al. (2024a) reported that all the major individual casein and whey components of colostrum sequentially decreased within the first 6 milkings postcalving. Colostrum is distinguished in particular by elevated levels of immunoglobulins, which provide critical passive immunity to the calf and make up over 70% of the total protein. Colostrum with an IgG (the predominant immunoglobulin in milk) content of ≥50 mg/mL is considered good quality (Kessler et al., 2021). McDermott et al. (2024a) reported that IgG decreased from 107 mg/mL in colostrum to 1.79 mg/mL from cows fed silage-based diets during the dry period. In contrast, Dunn et al. (2017a) recorded lower values in colostrum (average of 55 mg/mL IgG) from 21 grass-based dairy farms and emphasized the compositional variability in colostrum in terms of nutritional, immunological, and bacterial composition.

The fat content of colostrum is generally higher than that of mature milk (McGrath et al., 2016). In pasture-based systems, Dunn et al. (2017a), O'Callaghan et al. (2020), and McDermott et al. (2024b) reported values of 6.4%, 7.2%, and 5.8%, respectively, with decreases observed during subsequent milkings. Colostrum fat provides the energy source critical to the neonate in the hours after birth. During the transition from colostrum (from cows fed grass silage–based diets during the dry period) to mature milk, it has been reported that both total SFA and PUFA decrease with time, whereas an increase is observed in MUFA (O'Callaghan et al., 2020; McDermott et al., 2024b). These studies also reported decreases in palmitic acid (C16:0) and increases in oleic acid (C18:1 n-9), the 2 major fatty acids in milk, during the transition phase. O'Callaghan et al. (2020) suggested that milk produced after d 3 postparturition could be better for human consumption from a nutritional perspective due to lower levels of C16:0 and n-6 fatty acids along with concomitant increases in unsaturated fatty acids and conjugated linoleic acid. McDermott et al. (2024b) also provided, for the first time, quantitative data on the triglyceride composition (native molecular form of milk fatty acids) of colostrum from pasture-based cows. The distribution of medium- and high-molecular-weight triglycerides was affected significantly by parity, with implications for the thermal and material properties of milk fat.

In contrast with most of the other macroconstituents, lactose increases in concentration from colostrum to mature milk. Low lactose concentrations reflect the physiological needs of the calf, ensuring that colostrum is a concentrated source of proteins, including immunoglobulins. Bovine colostrum is also a rich source of oligosaccharides that act as prebiotics, boost gastrointestinal tract development, and promote calf immunity. Quinn et al. (2020) and McDermott et al. (2024a) reported significant decreases in total oligosaccharides in the days following birth, with the latter reporting values from 2.85 to 0.37 mg/mL in colostrum and the fifth transition milking, respectively.

Colostrum is also composed of a wide range of minor bioactive components including minerals, growth factors, enzymes, enzyme inhibitors, nucleic acids, cytokines, and vitamins (McGrath et al., 2016). McDermott et al. (2024c) reported that prepartum supplementation with selenium or rumen-protected choline to pasture-based cows did not affect the β-carotene concentrations of colostrum or transition milk. Pariyani et al. (2025) characterized the metabolic profile of colostrum from spring- and autumn-calving cows and highlighted the effects of seasonal, environmental, and dietary conditions on the development of bioactive components. Pasture-based colostrum and early transition milk contain numerous bioactive components beneficial to human health.

Compositional differences between colostrum from pasture-based or TMR-based systems are due predominantly to differences in diet during the dry period. In the case of pasture-based systems, this is typically ad libitum access to grass silage with supplementary concentrates and minerals. Dry period nutrition varies widely depending on geographical location and availability of feedstuffs. Dunn et al. (2017b) reported that concentrate supplementation in grass silage–based diets during an 8-week period before parturition had no effect on colostral IgG concentration, IgG yield, or fat, protein, and lactose contents compared with nonsupplemented grass silage. Concentrate supplementation during the dry period positively affects colostrum yield with the potential effect of diluting immunoglobulins (Conneely et al., 2013).

The narrow window of colostrum production in spring-calving cows offers an opportunity for collection and processing of colostrum (in excess to the needs of the calf) into high-value dairy ingredients and is a topic of growing research and commercial interest. However, logistical challenges remain with respect to collection, microbial stability, and processing of heat-sensitive bioactive ingredients.

Lactational effects are compounded by dietary changes in a pasture-based production system. However, considering the effect of a pasture-based diet relative to a conventional TMR-based diet in isolation, notable effects have been reported outside of the variability inherent to the lactation cycle. Regarding gross composition, variable effects have been observed. O'Callaghan et al. (2016) and Gulati et al. (2018) reported that milk from pasture-based diet exhibited significantly higher total solids (pasture mean range 14.0%–14.3%; TMR mean range 13.4%–13.8%), fat (pasture mean range 4.65%–4.85%; TMR mean range 4.39%–4.61%), protein (pasture mean range 3.65%–3.97%; TMR mean range 3.38%–3.66%), and casein (pasture mean range 2.95%–3.05%; TMR mean range 2.69%–2.84%) contents and significantly lower lactose (pasture mean range 4.72%–4.87%; TMR mean range 4.82%–4.89%) contents, which was also observed by Panthi et al., (2019; pasture mean 4.63%; TMR mean 4.74%) in comparison to milk derived from TMR. The composition of the TMR used by both O'Callaghan et al. (2016) and Gulati et al. (2018) consisted of (on a DM basis) 7.15 kg of grass silage, 7.15 kg of maize silage, and 8.3 kg of concentrates, including maize, maize distillers, molassed beet pulp, rapeseed meal, soybean meal, rolled barley, Megalac, salt, and mineral balancer.

Conflicting results were reported by Timlin et al., (2023), in which milk produced from a TMR system exhibited significantly higher total solids (pasture mean 13.6%; TMR mean 13.8%), protein (pasture mean 3.62%; TMR mean 3.72%), lactose (pasture mean 4.70%; TMR mean 4.81%), and proportional whey protein (pasture mean 15.5%; TMR mean 16.2%) in comparison to a grass-based system, highlighting the potential for variation in feed composition between different production years, as this study was carried out within the same farm infrastructure as the studies by O'Callaghan et al. (2016) and Gulati et al. (2018), albeit with the specific TMR being formulated based on the corresponding silage quality and composition for a given year. In this study, the TMR diet consisted of 9 kg of maize silage, 4.5 kg of grass silage, and 9 kg of concentrates on a DM basis. The composition of a TMR is not ubiquitous and can depend on a range of factors, including region, climate, resources, and provision of different ingredients, such as grass silage, maize silage, corn crop, spent brewers grain, beet pulp, barley, or soybean meal. The choice of ingredients will have distinctive effects on milk composition and quality, as highlighted by O'Callaghan et al. (2019), who compared the composition of milk samples from cows in a pasture system supplemented with crude parlor concentrate, palm kernel expeller plus parlor concentrate, soy hulls plus parlor concentrate, or molassed beet pulp plus parlor concentrate. The choice of feed variety led to variability in casein micelle size, acid-induced gel strength, and fatty acid and volatile profiles between the milk of each group.

Considering differences in the composition of pasture independent of TMR, a study by Kostovska et al., (2024), which compared perennial ryegrass (*Lolium perenne*) and multispecies sward pasture systems across 2 breeds of cow, identified an interactive effect of breed and diet on the total solids, fat, and lactose concentrations of milk, but did not identify a consistent effect of diet in isolation.

Concentrations of individual casein and whey proteins are not significantly altered by feeding systems (Magan et al., 2019). However, NPN content is an important component of the overall protein fraction, consistently demonstrating a dietary influence. Nonprotein nitrogen refers to the nitrogenous fraction of milk that remains soluble in 12% trichloroacetic acid (Rowland, 1938). It consists primarily of urea, ammonia, peptides, and free amino acids. By subtracting NPN from total milk nitrogen, the true protein nitrogen content can be determined. In bovine milk, NPN typically accounts for 5% to 6% of total milk nitrogen, with urea being the predominant component, representing ~50% to 74% of the total NPN fraction (Mehra et al., 1999; Gulati et al., 2019). Wolfschoon and Klostermeyer (1981) reported a slightly lower urea concentration in NPN at 48%. Milk urea nitrogen is widely recognized as an indicator of dietary protein supply and energy–protein balance in dairy cows (Zhao et al., 2025). However, several factors must be considered before evaluating the effects of diet on milk NPN content and composition. Pasture-based systems are inherently seasonal, relying on the grass growth cycle; therefore, variations related to lactation stage and forage availability can influence NPN levels. Furthermore, not all pasture systems are equivalent as the inclusion of mixed swards, such as clover or other legumes, can significantly affect both total NPN concentrations and the relative proportions of individual NPN components (e.g., urea).

Milk from grass and concentrate fed cows generally exhibits similar overall nitrogen composition. However, O'Callaghan et al. (2016) reported that cows fed a clover-based diet produced milk with significantly higher NPN (0.04% and 0.03%) levels compared with cows on grass-only or concentrate-based diets, with urea potentially accounting for up to 48% of total NPN. The composition of pasture, influenced by factors such as season, plant maturity, and soil fertility, can also contribute to fluctuations in milk NPN content. In the same study, O'Callaghan et al. (2016) observed notable seasonal variation in NPN concentrations, with levels remaining consistently higher in milk from cows grazing perennial ryegrass and white clover pastures throughout the year (ranging from 0.03% to 0.04%, wt/wt). This variability likely reflects changes in the stage of lactation typical of the Irish pasture-based production system. Similarly, Gulati et al. (2019) found that milk derived from TMR and white clover pasture diets contained higher NPN concentrations (5.54%–5.13%, expressed as a percentage of total nitrogen) than milk from cows fed perennial ryegrass (4.96%, expressed as a percentage of total nitrogen) during both mid and late lactation. In contrast, urea content was relatively consistent across all feeding systems, ranging from 35 to 58 mg/100 g.

The milk fat fraction is the most readily influenced by diet, which plays a crucial role in the fatty acid profile and the levels of health-promoting fats. Beyond nutritional composition, diet also affects the organoleptic properties of dairy products, affecting both their visual attributes and flavor profiles. As such, the influence of diet is most prominently reflected in butter, which remains the primary milk-fat-based consumer product exported and marketed globally.

Milk from grass-based diets has higher proportions of unsaturated fats and higher unsaturation (pasture mean 36.2; TMR mean 31.37), health-promoting (pasture mean 0.39; TMR mean 0.31), and desaturase (pasture mean 0.34; TMR mean 0.3) indices than milk from livestock fed TMR diets. The milk from grass-based diets also had lower levels of SFA (pasture mean 69.57; TMR mean 73.37 g/100 g of milk fat) and higher levels of MUFA (pasture mean 25.51; TMR mean 22.29 g/100 g of milk fat) and PUFA (pasture mean 4.98; TMR mean 4.34 g/100 g of milk fat), including higher levels of n-3 (pasture mean 0.79; TMR mean 0.37 g/100 g of milk fat) and CLA (pasture mean 1.47; TMR mean 0.61 g/100 g of milk fat) compared with indoor TMR feeding systems (Timlin et al., 2023).

A recent development in pasture systems has been the introduction of more diverse, multispecies swards (**MSS**), including plants such as chicory, plantain, meadow fescue, timothy, red and white clovers, and perennial ryegrasses. Although the introduction of these swards primarily focuses on improved nitrogen efficiency and reduced reliance on chemical fertilizer, there are also benefits in terms of drought resistance and improved biodiversity. Current research indicates that the impact of MSS on the gross macronutritional composition, yield, and functionality of milk is limited (Kostovska et al., 2024). However, there are interesting seasonal observations related to the fatty acid profile, particularly as a function of the stage of lactation, linked to changes in sward composition. Kostovska et al. (2025) reported increased proportions of PUFA (+12 to 45%) in mid and late lactation, whereas nutritionally beneficial C18:3 n-3 (1.06 vs. 0.73 g/100 g of milk fat) C18:2 n-6 (1.05 vs. 0.71 g/100 g of milk fat), and C22:5 n-3 (0.11 vs. 0.085 g/100 g of milk fat) PUFA were higher in mid and late lactation compared with early lactation periods for MSS diets and perennial ryegrass diets, respectively.

Milk minerals can be broadly classified into major and trace minerals. Numerous studies have investigated the effect of bovine diet on milk mineral composition, including those by Gulati et al. (2019) and Magan et al. (2026). Collectively, the literature suggests that diet has a greater influence on trace than on major minerals. For example, Magan et al. (2026) reported no significant differences in calcium, potassium, or sodium levels between skim milk powders derived from cows fed pasture-based versus TMR diets. In contrast, Gulati et al. (2018) observed higher concentrations of calcium and phosphorus in pasture-derived milk (142.2 and 104.0 mg/100 g, respectively) compared with TMR milk (131.8 and 101.0 mg/100 g, respectively), suggesting this was consistent with the higher casein content typically found in pasture-based samples. Although rates of silage feeding varied, the level of concentrate supplied in the TMR was consistent for the studies of Magan et al. (2026; 4.5 kg of grass silage, 9 kg of maize silage and 8.3 kg of concentrates on a DM basis) and Gulati et al. (2018; 7.15 kg of grass silage, 7.15 kg of maize silage, and 8.3 kg of concentrates on a DM basis).

Among the trace elements, iodine consistently emerges as one of the most diet-sensitive minerals. Magan et al. (2026) found markedly lower iodine concentrations in skim milk powders and corresponding whey samples from pasture-fed cows (~0.2 mg/100 g) compared with those from TMR-fed cows (~1.0 mg/100 g). Similarly, O'Brien et al. (2013) demonstrated that both dietary and environmental factors influence iodine transfer into milk. In their study, dietary supplementation of iodine (70 mg/d) increased milk iodine concentrations from approximately 449 to 915 µg/kg. However, iodine-based teat disinfectants can also elevate milk iodine levels. Elevated iodine concentrations are of particular concern for the infant milk formula industry, where excessive iodine may result in noncompliance with CODEX specifications for mineral composition in incoming skim milk. Supporting these findings, Gulati et al. (2019) also reported substantially higher iodine levels in milk from TMR-fed cows (4,746–6,390 µg/kg) compared with those from pasture-based systems (230–616 µg/kg). Other trace minerals show similar diet-related trends: Magan et al. (2026) reported significantly lower selenium (pasture mean 0.017 mg/100 g; TMR mean 0.034 mg/100 g) and iodine (pasture mean 0.222 mg/100 g; TMR mean 0.980 mg/100 g) concentrations but higher manganese (pasture mean 0.049 mg/100 g; TMR mean 0.015 mg/100 g) levels in milk obtained from pasture-based systems compared with TMR-fed cows.

During the analysis of milk fat from pasture and indoor feeding systems (Zhou et al., 2025), significantly higher (*P* < 0.05) levels of the fat-soluble forms of vitamin K were observed in milk from pasture, as a function of both diet and stage of lactation. Although Timlin et al. (2021) stated that feed influences the fat-soluble vitamins in milk, with the exception of vitamin K, it is noteworthy that the content of vitamin D in milk has been reported to be 4 times higher in milk produced in the summer compared with winter (Kurmann and Indyk, 1994). The nature of pasture grazing compared with indoor systems likely warrants additional research to clarify any advantages associated with the beneficial effects of solar exposure on vitamin D levels, which are attributable to grazing systems.

Timlin et al. (2024b) conducted a further study investigating fat-soluble nutritional components in milk from pasture-based and TMR-based diets, observing significantly greater concentrations of β-carotene, lutein, retinol (vitamin A), α-tocopherol (vitamin E), and zeaxanthin in milk derived from pasture. Analysis of water-soluble B vitamins in skim milk and whey from pasture and TMR diets also identified notable differences between these systems (Magan et al., 2020). Concentrations of thiamine (B_1_), riboflavin (B_2_), and biotin (B_7_) were significantly higher in pasture-derived samples, whereas the TMR-derived samples exhibited significantly higher concentrations of niacin (B_3_).

The most evident difference in organoleptic perception between dairy products derived from pasture or non-pasture-based diets is visual. Grass-derived products exhibit a characteristic golden hue, due primarily to significantly higher concentrations of β-carotene (Timlin et al., 2024b), although riboflavin, which has been shown to be present in concentrations more than twice as high in pasture-derived products relative to TMR-derived products, also contributes a pigmentation effect (Magan et al., 2020). This observation has consistently been quantified and observed throughout numerous studies across liquid milk, butter, cheese, and whole milk powder products (Couvreur et al., 2006; Nozière et al., 2006; O'Callaghan et al., 2017; Faulkner et al., 2018; Magan et al., 2019; Timlin et al., 2024b).

Texture, color, and flavor are the other primary quality attributes of butter, which are routinely used to establish a point-of-difference between pasture and non-pasture-derived products. Textural variability primarily arises due to the relative concentrations of and ratio between palmitic acid (C16:0) and oleic acid (C18:1) in milk fat used for butter manufacture. Palmitic acid is a saturated, high-melting-point fatty acid that tends to be present in significantly higher concentrations in milk from a TMR system relative to that from pasture-based systems, which are associated with significantly higher oleic acid, PUFA, and n-3 fatty acid contents (Couvreur et al., 2006; O'Callaghan et al., 2016; Timlin et al., 2024a). Palmitic acid content has also been associated with textural variation in cheddar cheese, consistent with the trend observed for butter (O'Callaghan et al., 2017).

Regarding flavor and the volatile compounds on which it depends, significant variation has also been demonstrated between pasture-derived and conventionally produced milk and butter samples. Although local and cross-cultural sensory paneling studies with both consumer and trained panelists have indicated that panelists tend to show preference for samples produced from the system with which they are most familiar, and differences in sensory perception exist between cultures, sensory attributes and volatile profiles have been shown to be significantly influenced by feeding system (O'Callaghan et al., 2016; Faulkner et al., 2018; Cheng et al., 2020). Volatile compounds which have been significantly associated with pasture feeding include dimethyl sulfone, which has a low odor threshold for sensory perception and has been associated with a “cooked milk” flavor, and toluene, a metabolic product of β-carotene (Clarke et al., 2019), whereas carbohydrate esters and aldehydes have been associated with milk from TMR-based systems (Faulkner et al., 2018). *p*-Cresol was also identified by Faulkner et al. (2018) as significantly associated with white clover inclusion in pasture and as the compound responsible for the “barnyard” aroma assigned to pasture-derived milk products, representing a potential biomarker for grass-fed products.

Pasture-derived or grass-fed dairy products often demand a premium price in international markets. This is in part attributed to consumers perceptions of such products and an association with healthiness and naturalness, as demonstrated by McGuinness et al. (2022) in cheese products. Some of these perceptions appear to have a basis in fact, specifically in relation to the beneficial effects pasture-based feeding has on the fatty acid profile and organoleptic properties of milk and dairy products, as discussed previously. Despite the growing market demand for grass-fed dairy, there remains an ongoing challenge surrounding development of testing methods and classification criteria for verification of pasture-derived milk and dairy products; this, in part, is related to certain ambiguity across countries and within regions as to what farm-level factors define a grass-fed dairy system. These can range from the proportion of grass in the cow diet to the number of days or hours with access to pasture, all of which are highly dependent on regional climatic conditions, natural resources, and ability to grow grass.

Nevertheless, the development of such methods could be beneficial in defining the provenance surrounding grass-fed status in order to increase consumer confidence in purchasing grass-fed products. In more recent years, significant efforts have been made to develop methods and identify potential biomarkers that could be used for testing and classifying milk as grass-fed. Considering the composition of milk the fat fraction is the most variable and most affected by the cow diet, with longer chain non-de novo synthesized fatty acids being directly influenced by dietary intake, several studies have highlighted significantly increased concentrations of fatty acids associated with pasture intake. The most prominent of these include rumenic acid (CLA *cis*-9,*trans*-11), α-linolenic acid, and n-3 fatty acids, whereas palmitic acid levels and n-6 fatty acids have been associated with TMR-derived milk (O'Callaghan et al., 2016), as such Capuano et al. (2014b) concluded using partial least squares discriminant analysis models of milk fatty acids, the inclusion of fresh grass in the cow diet can be accurately predicted. In agreement with research by Couvreur et al. (2006), Timlin et al. (2023) demonstrated the linear response to milk fatty acids with the proportion of pasture in the cow diet, using receiver operating characteristic (**ROC**) curve analysis and identified 3 fatty acids as excellent biomarkers (ROC >0.9) between pasture feeding and TMR with C18:3n-3, CLA C18:2 *cis*-9,*trans*-11, and C18:2n-6 *cis*.

Timlin et al. (2024b) demonstrated how high proportions of pasture in the cow diet resulted in a 2-fold increase in β-carotene on average across lactation. A biomarker model in this case was developed based on fat-soluble compounds β-carotene, retinol, lutein, zeaxanthin, and α-tocopherol demonstrating good and excellent (ROC >0.85) differentiation of pasture-derived milk and TMR-derived milk. More recently metabolomics has been further explored to identify biomarker compounds in pasture-derived milk with Rojas-Gómez et al. (2025) most notably highlighting hippurate as a robust marker of pasture-based feeding, in agreement with other studies from goat milk (Carpio et al., 2013). Collectively across fatty acids, fat-soluble compounds, and milk metabolites, there exists significant potential to develop methods for the verification of grass-fed status, although challenges remain because in some cases, methods will need to be tailored to region-specific grass-fed standards that would set pass/fail criteria, requiring large datasets for validation. However, these tests require time and expensive equipment, and as such, rapid type analysis methods would be very beneficial for high-throughput screening of milk.

With this in mind, mid-infrared technologies are widely applied to milk samples for rapid composition and quality analysis of milk. Capuano et al. (2014a) developed a method for the prediction of grass-based feeding of cows using Fourier-transform infrared spectroscopy coupled with partial least squares discriminant analysis classification models. More recently, Frizzarin et al. (2021) explored a series of machine learning methods applied to milk mid-infrared spectra for the discrimination of grass-fed versus non-grass-fed milk across >4,000 samples, developing a method with high accuracy for the prediction of cow diet and identifying spectral regions that correspond to cow diet. In the future, this could provide rapid screening and validation of grass-fed dairy. Further research is warranted on the development of robust rapid screening of milk to support grass-fed status and standards, where mid-infrared approaches could be used in conjunction with more advanced chromatographic approaches to quantify the selected biomarkers mentioned previously.
